# Novel High‐Resolution Lipidomes Could Serve as New Biomarkers for Diabetic Retinopathy: A Bidirectional and Mediated Mendelian Randomization Study

**DOI:** 10.1111/jcmm.70614

**Published:** 2025-06-04

**Authors:** Yuxin Sun, Ziran Zhang, Zejun Chen, Zhengran Li, Zijin Wang, Fanye Wu, Xinyu Ma, Shaoyu Wang, Mingzhe Cao, Guoguo Yi, Min Fu

**Affiliations:** ^1^ The Second School of Clinical Medicine Southern Medical University Guangzhou China; ^2^ Ophthalmology Department Zhujiang Hospital Affiliated With Southern Medical University Guangzhou China; ^3^ Department of Ophthalmology, the Seventh Affiliated Hospital Sun Yat‐Sen University Shenzhen China; ^4^ Department of Ophthalmology, the Sixth Affiliated Hospital Sun Yat‐Sen University Guangzhou China; ^5^ Biomedical Innovation Center, the Sixth Affiliated Hospital Sun Yat‐Sen University Guangzhou China; ^6^ Center for Teaching and Learning Development Southern Medical University Guangzhou China

**Keywords:** circulating inflammatory protein, diabetic retinopathy, interleukin‐10, lipidome, mediation analysis, Mendelian randomization, shotgun lipidomics technique, triacylglycerol

## Abstract

Although lipid metabolism is a critical factor in the pathogenesis of diabetic retinopathy (DR), the connection between lipidome and DR is still a subject of debate. We aimed to demonstrate that lipidome could serve as novel biomarkers for DR and elucidate the mediating role of inflammatory factors. Data for our investigation are available from the GWAS catalogue and FinnGen Biobank. The bidirectional Mendelian randomization (MR) analyses were conducted to assess the “total effect” between lipidome and DR and its subtypes. Subsequently, the mediation analyses were performed to explore the involvement of circulating inflammatory proteins in mediating the connection between them. Mediation proportion was calculated to measure the contribution of inflammatory factors to the overall effect. Ultimately, a battery of sensitivity tests proceeded to examine the dependability of the findings. This study has revealed a causal relationship between lipidome and different stages of DR. Additionally, we have successfully discovered a range of new lipids that protect against DR and have the potential to serve as new markers. This study also highlights the important role of inflammatory factors in elucidating the protective mechanisms of lipids against DR and provides new perspectives on lipidomic‐based treatments and cytokine‐targeted interventions for DR.

**Abbreviations**
β‐NGF: beta‐nerve growth factor
Bp: Base pairs
BWMR: Bayesian weighted MR
Cer: Ceramide
CI: Confidence interval
DG: Diacylglycerol
DM: Diabetes mellitus
DR: Diabetic retinopathy
EWAS: Epigenome‐wide association study
FGF‐19: Fibroblast growth factor‐19
GWAS: Genome‐Wide Association Study
HbA1c: Glycosylated haemoglobin, type A1C
HDL‐C: High‐density lipoprotein cholesterol
IL: Interleukin
IVs: Instrumental variables
IVW: Inverse variance weighted
LAP TGF‐β‐1: Latency‐associated peptide transforming growth factor beta 1
LD: Linkage disequilibrium
LDL‐C: Low‐density lipoprotein cholesterol
LTA: Lymphotoxin‐alpha
MR: Mendelian randomization
MR‐PRESSO: MR Pleiotropy RESidual Sum and Outlier
NKT: Natural killer T
NPDR: Non‐proliferative diabetic retinopathy
OR: Odds ratio
PC: Phosphatidylcholine
PDR: Proliferative diabetic retinopathy
PE: Phosphatidylethanolamine
SD: Standard deviation
Se: Standard error
SE: Sterol ester
SIRT2: Sirtuin 2
SLAMF1: Signalling lymphocytic activation molecule family member 1
SM: Sphingomyelin
SNP: Single nucleotide polymorphism
TC: Total cholesterol
TG: Triglyceride
TNFB: Tumour necrosis factor beta

## 1 Introduction

Diabetes mellitus (DM) represents a mounting global health crisis, with its prevalence among adults aged 20–79 projected to rise from 536.6 million (10.5%) in 2021 to 783.2 million (12.2%) by 2045 [1]. As the most prevalent microvascular complication of DM, diabetic retinopathy (DR) remains the leading cause of preventable blindness in working‐age populations, affecting 34.6% of diabetic patients [2]. Clinically categorised into non‐proliferative (NPDR) and proliferative (PDR) stages [3], DR progression underscores a critical therapeutic paradox: while early intervention reduces vision loss, current clinical strategies predominantly target advanced stages with limited efficacy. This discrepancy highlights the urgent need to identify stage‐specific risk factors and develop precision interventions to mitigate retinal damage.

Numerous pieces of evidence reveal that lipid metabolism disorders contribute significantly to the development of DR [4]. Conventional lipid profiling, relying on total cholesterol (TC), triglycerides (TG), low‐density lipoprotein (LDL‐C), and high‐density lipoprotein (HDL‐C) measurements [5], has yielded inconsistent associations with DR in observational studies. These biases likely arise from confounding factors, reverse causality, and inadequate sample sizes, compounded by the complexity of lipid species involved in the development of DR. Crucially, traditional lipid parameters lack the resolution to detect early metabolic shifts for predicting DR.

With the application of modern and efficient lipidomics technologies (like mass spectrometry and shotgun lipidomics technique), one can perform high‐throughput and more precise analysis of the composition and expression levels of lipids, defining lipid molecules into species or subspecies according to the ratio of carbon to hydrogen, as well as the type, number, and position of chemical groups [6]. Plasma lipidome provides information about lipid properties, metabolic processes, and biological functions currently unavailable from conventional clinical lipid chemistry. It can be used to elucidate the underlying mechanisms of numerous complex disease processes. At present, lipidome has been employed in the investigation of DR and has identified the potential of lipids such as ceramide (Cer), sphingomyelin (SM), and phosphatidylcholine (PC) as biomarkers of DR, thus expanding the understanding of the mechanisms of microvascular complications in diabetes [7]. Although several studies have elucidated that specific human serum lipids have an essential role in the progression of DR, the exact underlying mechanism is unclear, and the existing studies suffer from the drawbacks of insufficient sample size and the influence of interfering factors and inverse causality.

Mendelian randomization (MR) analysis uses genetic variation as instrumental variables (IVs) to infer causality [8]. Unlike conventional observational analyses, MR analyses are less prone to bias or confounders. They can provide robust empirical evidence for studying the relationship between lipidome and DR. In this study, we performed bidirectional MR analyses based on genome‐wide association study (GWAS) data, incorporating 179 lipid species and different types of DRs (NPDR and PDR). We aimed to demonstrate that lipidome could serve as novel biomarkers for DR. Furthermore, systemic inflammation as a key driver of DR [9] and its mediating role in this relationship emphasises the critical role of inflammatory factors in elucidating the protective mechanisms of lipidome against DR. It provides new perspectives on lipidomic‐based treatments and cytokine‐targeted interventions for DR.

## 2 Materials and Methods

### 2.1 Research Design

**FIGURE 1:**
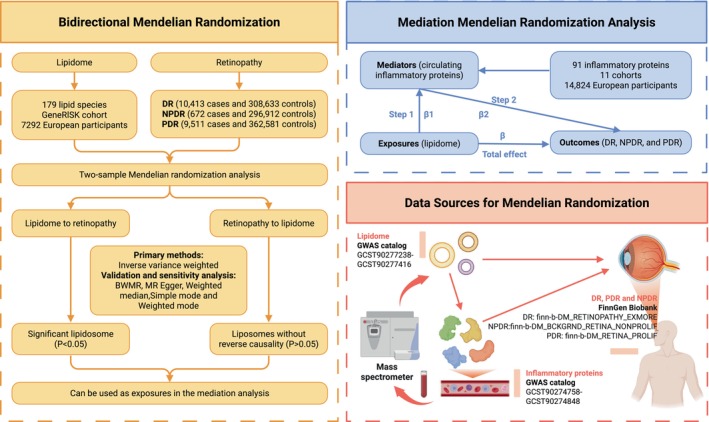
The design and flow chart of the investigation. The research aimed to determine whether circulating proteins of inflammation (mediators) play a causal role in mediating the effects of lipidome (exposures) on DR, NPDR, and PDR (outcomes). Initially, a two‐sample bidirectional MR analysis was performed to investigate the causal relationship between lipidome and retinopathy. The mediated MR used a two‐step approach: the first step was to determine the causal relationship between lipids and inflammatory proteins in the blood. The next step is to assess the causative influence of circulating inflammatory proteins on DR, NPDR, and PDR.

We applied a two‐way MR framework to examine potential bidirectional causal connections between lipidome and DR and its subtypes (PDR and NPDR). In order to enhance our comprehension of the impacts mediated by circulating inflammatory proteins, we adopted a two‐step methodology for conducting mediation MR (Tables S1–S4) analysis. Figure 1 displays the design and course of the investigation.

### 2.2 Data Source

The GWAS data for 179 lipid species came from a study conducted on the GeneRISK cohort, which consisted of 7174 European participants. The original paper provides a thorough description of the naming conventions and measurement methodologies used for the lipid species [6]. The statistics can be downloaded using the accession numbers GCST90277238‐GCST90277416 from the GWAS catalogue [10]. Lipid species in this study followed the lipid identifier of the SwissLipids database [11, 12].

GWAS summary statistics for DR (308,633 controls and 10,413 cases) and PDR (362,581 controls and 9511 cases) were extracted from the ninth version of the FinnGen Biobank [13], which contains genotype and phenotype data from 377,277 (210,870 women and 166,407 men) Europeans. GWAS summary data of NPDR (672 cases and 296,912 controls) were acquired from the seventh version of the FinnGen Biobank [14] with a total sample size of 309,154 (173,746 women and 135,408 men).

Genetic data for 91 circulating inflammatory proteins were derived from Zhao's GWAS meta‐analysis based on 11 cohorts (14,824 European participants) and were downloaded from the GWAS catalogue [10] (accession codes GCST90274758–GCST90274848). The initial publication included comprehensive techniques for quantifying quantities of circulating proteins.

This analysis utilised publicly accessible or published GWAS pooled datasets. The original study had already received ethical permission, so no extra ethical approval was necessary.

### 2.3 Selection of Instrumental Variables

In MR analysis, we utilised single nucleotide polymorphisms (SNPs) as genetic IVs to examine the causality between lipidome and DR at the gene level. IVs must share a connection with the exposure variable but not with the outcome variable. Additionally, they should not be related to any known confounders, such as those driven by diabetes [8]. We identified appropriate IVs by following the subsequent procedures. Initially, we extracted SNPs that were significantly correlated with exposure at the whole‐genome level (*p* < 5 × 10^−8^). Subsequently, we employed linkage disequilibrium (LD) clustering to acquire independent IVs (*r* ^2^ < 0.001) within a distance of 10,000 base pairs (bp) [15]. Thirdly, we selected robust IVs (*F* > 10) for MR analysis to minimise the influence of mild instrumental bias [16]. Finally, to address diabetes‐related confounding, we systematically screened these IVs through the LDlink web tool for pleiotropic associations with diabetic traits (HbA1c, glucose, DM status) [17] and excluded SNPs linked to other diabetic complications at genome‐wide significance (*p* < 1 × 10^−5^) using PhenoScanner V2 [18]. The retained SNPs, demonstrating no significant associations with diabetes‐related confounders, were subsequently employed as validated IVs in our MR framework to ensure diabetes‐independent causal inference between lipidome and DR progression.

### 2.4 Bidirectional MR

To estimate the causal correlation between lipidome and DR, NPDR, and PDR, we performed two‐sample bidirectional MR analyses. This relationship is commonly known as the “total effect” and is symbolised by *β*. Inverse variance weighted (IVW) was the primary analysis method for random effects [19], complemented by Bayesian‐weighted Mendelian randomization (BWMR), MR‐Egger, weighted median, simple mode, and weighted mode methods for validation [20], with all results systematically documented. Lipids eligible for mediation analysis were required to meet stringent criteria demonstrating (1) established forward causal associations and (2) the absence of reverse causation.

### 2.5 Mediation Analysis

We undertook a two‐stage MR analysis to examine how circulating inflammatory factors connect the lipidome to DR and its subtypes (NPDR and PDR). The indirect impacts (mediation effects) of inflammatory factors were determined by computing the product of *β*1 and *β*2. *β*1 indicates the effect of lipids on inflammatory factors, while *β*2 denotes the impact of inflammatory factors on DR, NPDR, and PDR [21]. The mediation proportion was the ratio of the mediation effect to the full effect. This allows us to measure the contribution of inflammatory variables to the overall effect. In addition, we calculated the two‐tailed P‐values using the Delta method.

### 2.6 Sensitivity Analysis

Initially, we carried out Cochran's Q test to assess the variability in each of the SNPs [22]. The IVW random‐effects model accounted for heterogeneity (*p* < 0.05), while the fixed‐effects model was employed when heterogeneity was absent. Furthermore, the MR‐Egger regression analysis provided MR estimates, and their intercept elements detected potential horizontal pleiotropy. The intercepts showed a deviation from zero (intercept *p* < 0.05), indicating the existence of horizontal pleiotropy [23]. Subsequently, MR‐PRESSO was used to detect multiple instances, identify significant outliers, and address potential confounders by removing them [24]. Ultimately, the weighted median [25], weighted mode [26], and sample mode were applied to evaluate the reliability and coherence of the findings as supplementary validations.

The TwoSampleMR software package (version 0.6.2) for R (version 4.3.2) was used for MR analyses and sensitivity analyses. The level of statistical significance was set at *p* < 0.05.

## 3 Results

### 3.1 Genetic Causality Between Lipidome and DR and Its Subtypes (NPDR and PDR)

**FIGURE 2:**
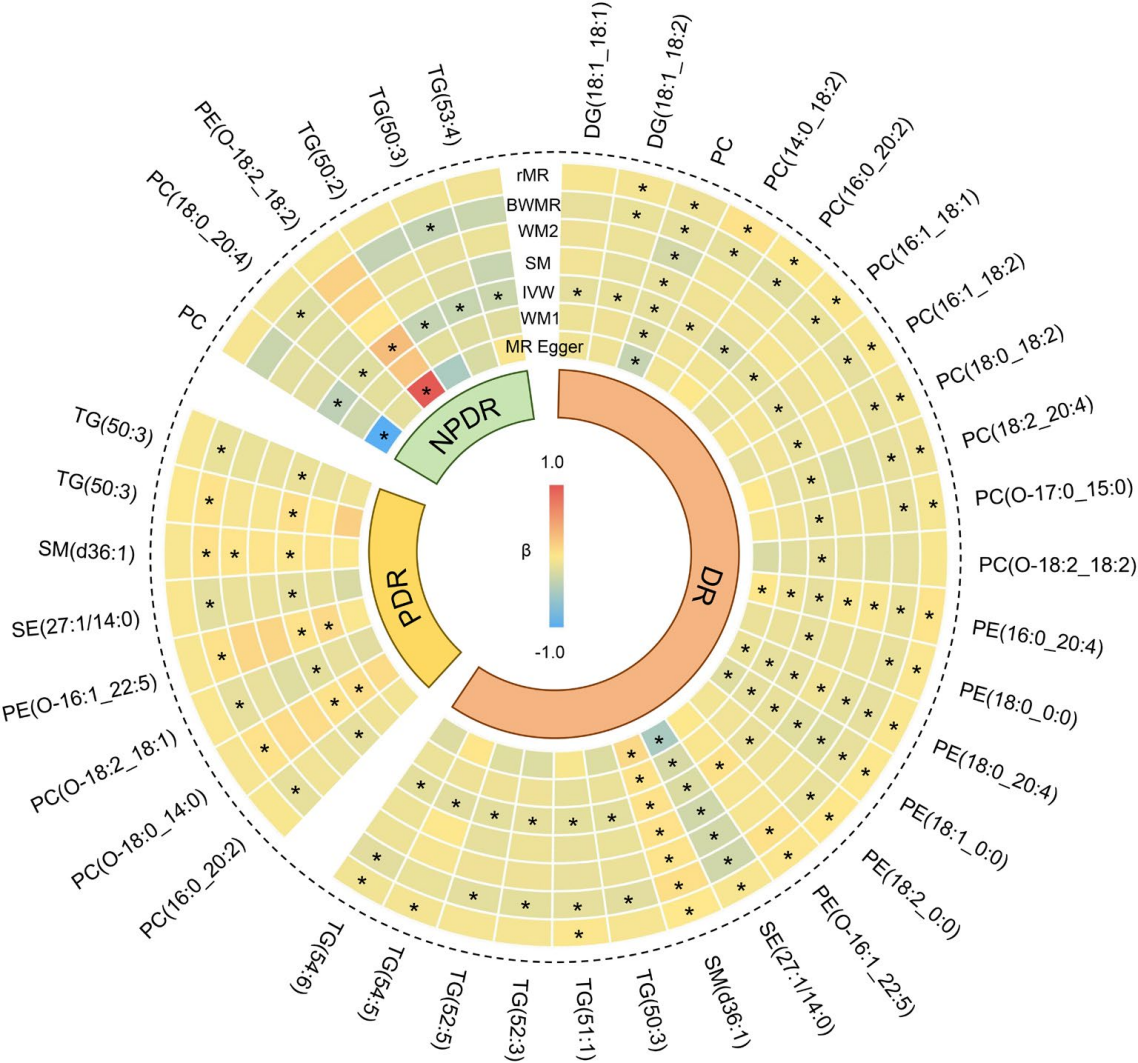
A heatmap displays bidirectional causality between lipidome and DR, NPDR, and PDR. Statistical significance is indicated by an asterisk (*) for a *p*‐value of < 0.05. The cell's colour represents the *β* value linked to the pair, with red indicating positive values and blue signifying negative values. BWMR, Bayesian weighted Mendelian randomization; DG, Diacylglycerol; IVW, inverse variance weighted; PC, Phosphatidylcholine; PE, Phosphatidylethanolamine; rMR, reverse Mendelian randomization; SE, Sterol ester; SM, simple mode; SM, Sphingomyelin; TG, Triglyceride; WM1, weighted median; WM2, weighted mode.

The bidirectional causal relationships between lipidome and DR, NPDR, and PDR are illustrated in Figure 2 (*p < 0.05). Using IVW as the primary analytical approach with BWMR, MR‐Egger, weighted median, simple mode, and weighted mode for sensitivity analyses, we systematically validated these associations. Comprehensive results are detailed in Tables S1–S3.

In evaluating lipidome‐DR causality, 22 lipid species demonstrated statistical significance across both IVW and BWMR methods. Reverse MR analysis revealed three lipids—TG (50:3) (*p* = 0.149), TG (52:3) (*p* = 0.149), and TG (52:5) (*p* = 0.149) – lacking reverse causation. However, they exhibited protective effects against DR risk, specifically TG (50:3) (OR 0.894, 95% CI [0.834–0.959], *p* 0.002), TG (52:3) (OR 0.907, 95% CI [0.844–0.974], *p* 0.007), and TG (52:5) (OR 0.894, 95% CI [0.823–0.969], *p* 0.007). These 3 lipids qualified for subsequent mediation analysis for DR by satisfying criteria requiring confirmed unidirectional causality (forward association without reverse effects).

Forward MR revealed 2 lipid species significantly causally associated with NPDR. They were PC (18:0_20:4) (OR 0.873, 95% CI [0.765–0.997], *p* 0.045) and TG (50:3) (OR 0.753, 95% CI [0.599–0.947], *p* 0.015). The reverse MR found that the genetic predisposition to NPDR had no impact on either PC (18:0_20:4) or TG (50:3). So it is feasible to use these 2 lipids as exposures for mediation MR of NPDR.

Similarly, the SE (27:1/14:0), PE (O‐16:1_22:5), PC (O‐18:0_14:0), PC (16:0_20:2), PC (O‐18:2_18:1), SM (d36:1), TG (46:2), and TG (50:3) obviously reduced the incidence of PDR without reverse causality and were candidate exposures for mediation analysis of PDR. Interestingly, TG (50:3) was the only lipid that showed an outstanding causal relationship with all of DR, NPDR, and PDR.

### 3.2 Mediation Analyses of Potential Circulating Inflammatory Proteins

#### 3.2.1 Step 1: Causal Effects of Lipidome on Mediators

**FIGURE 3:**
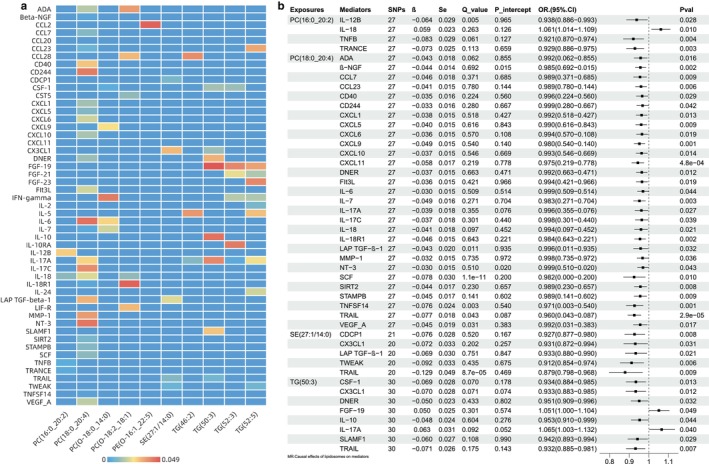
Causal effects of lipidome on mediators. (a) Heat map illustrating the causative impact of lipids on the levels of inflammatory proteins in the bloodstream. The cell's colour signifies the *p*‐value relating to the pairing, and the blue colour corresponding to a value of 0 indicates the absence of the causal relationship. (b) Forest plot showing the causality of lipidome on circulating inflammatory proteins using the IVW method. The IVW‐derived OR and 95% CI have been rigorously validated through four complementary analytical approaches (MR‐Egger, weighted median, simple mode, and weighted mode), with full verification details provided in Table S4. *β*, genetic effect sizes; CI, confidence interval; OR, odds ratio; *p*val, *p* value; Q_value, *p* value for Cochran's Q test; Se, standard error; SNPs, single nucleotide polymorphisms.

Circulating inflammatory proteins with significant causal associations with selected lipids are shown in Figure 3a. Figure 3b demonstrates significant causal relationships between lipids and circulating inflammatory proteins analysed using the IVW method. To ensure methodological rigour, we further implemented four complementary approaches (MR‐Egger, weighted median, simple mode, and weighted mode) for causal validation. The consolidated results, encompassing verification of the OR values and 95% CI depicted in Figure 3b, are comprehensively documented in Table S4. As shown in the figure, lipids were negatively correlated with most inflammatory factors. Nevertheless, the PC (16:0_20:2) exhibited an uptrend with interleukin‐18 (IL‐18, OR 1.061, 95% CI [1.014–1.109], *p* 0.010) among the factors. In addition, the level of TG (50:3) showed a positive correlation with fibroblast growth factor‐19 (FGF‐19, OR 1.051, 95% CI [1.000–1.104], *p* 0.049) and IL‐17A (OR 1.065, 95% CI [1.003–1.132], *p* 0.040).

#### 3.2.2 Step 2: Causal Effects of Possible Mediators on DR, NPDR, and PDR

**FIGURE 4:**
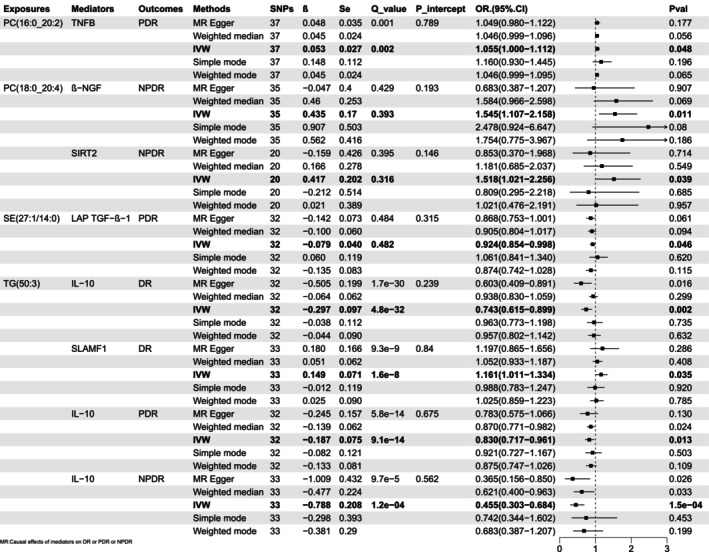
Forest plot showing the causality of inflammatory proteins (mediators) on outcomes. CI, confidence interval; DR, diabetic retinopathy; IVW, inverse variance weighted; NPDR, non‐proliferative diabetic retinopathy; OR, odds ratio; PDR, proliferative diabetic retinopathy; *p*val, *p* value; Q_value, *p* value for Cochran's Q test; Se, standard error; SNPs, single nucleotide polymorphisms; *β*, genetic effect sizes.

Based on the aforementioned results of inflammatory factors having substantial causal correlations with lipids, we further analysed the influence of these inflammatory factors on the outcomes (DR, NPDR, and PDR). The combinations with significant causal correlations are displayed in Figure 4. Surprisingly, IL‐10 was significantly and causally associated with DR (OR 0.743, 95% CI [0.615–0.899], *p* 0.002), NPDR (OR 0.455, 95% CI [0.303–0.684], *p* 1.5e‐04) when TG (50:3), and PDR (OR 0.830, 95% CI [0.717–0.961], *p* 0.013) was the exposure. In other words, the probabilities of developing DR were reduced by 25.7%, NPDR by 54.5%, and PDR by 17.0% for every one standard deviation (SD) rise in IL‐10. As demonstrated, IL‐10 has the greatest effect on NPDR, followed by DR and finally PDR.

In particular, signalling lymphocytic activation molecule family member 1 (SLAMF1) had a causal influence limited to DR (OR 1.161, 95% CI [1.011–1.334], *p* 0.035). Similarly, beta‐nerve growth factor (β‐NGF, OR 1.545, 95% CI [1.107–2.158], *p* 0.011) and Sirtuin 2 (SIRT2, OR 1.518, 95% CI [1.021–2.256], *p* 0.039) had substantial causative impacts on the NPDR only, and tumour necrosis factor beta (TNFB, OR 1.055, 95% CI [1.000–1.112], *p* 0.048) and the latency‐associated peptide transforming growth factor beta 1 (LAP TGF‐β‐1, OR 0.924, 95% CI [0.854–0.998], *p* 0.046) were significantly causally linked to the PDR only.

### 3.3 Mediation Analysis

**FIGURE 5:**
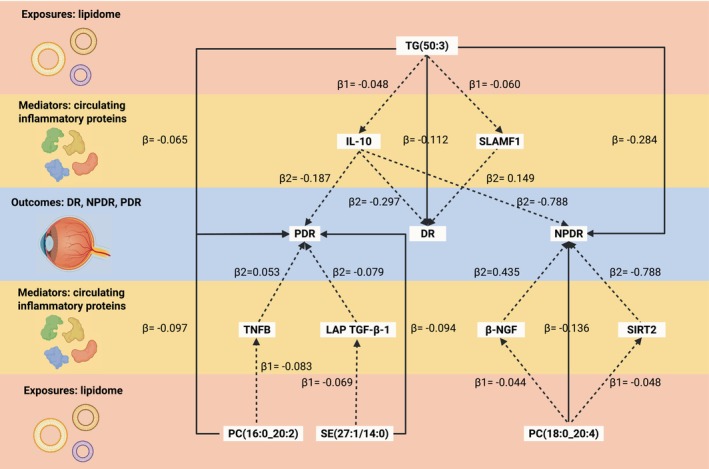
Mediation analysis of two‐step Mendelian randomization. The diagram illustrates the involvement of circulating inflammatory proteins in mediating the causative effect of exposures on outcomes. *β*, the total causal effect of exposures with outcomes; *β*1, the effect of exposures on mediators; *β*2, the impact of mediators on outcomes; DR, diabetic retinopathy; NPDR, non‐proliferative diabetic retinopathy; PDR, proliferative diabetic retinopathy.

Figure 5 displayed four lipids mediating their effects on DR, NPDR, and PDR through 6 inflammatory factors. We additionally calculated the mediation effects (*β*12 = *β*1 × *β*2), the direct effect (*β*_dir = *β*—*β*12), and the mediation proportion (*β*12_*p* = *β*12/*β*), as shown in Table 1. The proportion mediated by SLAMF1 was 8.00%. β‐NGF accounted for a 14.00% influence, and SIRT2 accounted for a 13.50% effect of the PC (18:0_20:4) on NPDR. TNFB mediated a 4.50% effect of PC (16:0_20:2) on PDR.

**TABLE 1:** Quantitative analysis of mediation effects.

Exposures	Mediators	Outcomes	*β*	*β*1	*β*2	*β*12	*β*_dir	*β*12_*p*	*p*
PC (18:0_20:4)	β‐NGF	NPDR	−0.136	−0.044	0.435	−0.019	−0.117	14.00%	0.622
	SIRT2	NPDR	−0.136	−0.044	0.417	−0.018	−0.118	13.50%	0.404
PC (16:0_20:2)	TNFB	PDR	−0.097	−0.083	0.053	−0.004	−0.093	4.50%	0.146
SE (27:1/14:0)	LAP TGF‐β‐1	PDR	−0.094	−0.069	−0.079	0.005	−0.099	—	0.111
TG (50:3)	IL‐10	DR	−0.112	−0.048	−0.297	0.014	−0.126	—	0.524
	SLAMF1	DR	−0.112	−0.060	0.149	−0.009	−0.103	8.00%	0.798
	IL‐10	NPDR	−0.284	−0.048	−0.788	0.038	−0.322	—	0.828
	IL‐10	PDR	−0.065	−0.048	−0.187	0.009	−0.074	—	0.818

Abbreviations: *β*, the total causal effect between exposures and outcomes; *β*_dir, the direct effect; *β*1, the effect of exposures on mediators; *β*12, the mediation effects; *β*12_p, the mediation proportion; *β*2, the impact of mediators on outcomes; *β*‐NGF, beta‐nerve growth factor; TNFB, tumour necrosis factor beta; DR, diabetic retinopathy; IL‐10, interleukin‐10; LAP TGF‐β‐1, latency‐associated peptide transforming growth factor beta 1; NPDR, non‐proliferative diabetic retinopathy; *p*, two‐tailed *p*‐values calculated using the Delta method; PC, phosphatidylcholine; PDR, proliferative diabetic retinopathy; SE, sterol ester; SIRT2, sirtuin 2; SLAMF1, signalling lymphocytic activation molecule family member; TG, triacylglycerol.

### 3.4 Sensitivity Analysis

We used multiple methods to test for heterogeneity and multiplicity of analyses. All sensitivity analysis results in this study are presented in Tables S1–S3. Although we found heterogeneity and pleiotropy in some results, they are acceptable.

## 4 Discussion

Recent studies have shown that PC, PE, and Cer are the most significantly modified lipid molecules in the DR, whereas TG, PC, Cer, and SM are the most modified lipids in the PDR [27]. In this study, we revealed a causal relationship between the lipidome and different stages of DR, where elevated levels of TG (50:3) were associated with a reduced risk of both NPDR and PDR, an effect that could be mediated through IL‐10. In different subtypes of DR, we found that PC (18:0_20:4) could exert a protective effect against NPDR through the mediating effects of β‐NGF and SIRT2, whereas in PDR PC (16:0_20:2) and SE (27:1/14:0) exerted a protective effect through the mediating effects of TNFB and LAP TGF‐β, respectively. The clinical significance of this study lies in the discovery of the potential of new lipids as biomarkers. This serves as a basis for the staging of DR. It highlights that cellular inflammatory factors could be potential therapeutic targets for DR. This provides the opportunity for personalised treatment protocols and guidance for different stages of DR.

Our research indicates that there is a connection between SM and DR, which is consistent with a previous MR study [28]. An increase in SM (d36:1) significantly increases the risk of developing DR (*p* < 0.05). SM is a key component of retinal lipids, and its activation can lead to various biological effects that ultimately contribute to the progression of DR. For instance, the hydrolysis of SM to Cer by acidic sphingomyelinases will enhance receptor aggregation, amplifying inflammatory signalling via several receptors (e.g., IL‐1β and TNF‐α) that mediate common pathological changes in DR [29, 30]. Furthermore, the SM pathway is involved in the combined action of lipopolysaccharide and palmitate to increase the expression of proinflammatory factors and promote apoptosis in retinal neuronal cells [31].

Numerous studies have identified distinct lipidomic profiles in DR [32, 33]. Conventional lipidomic studies have shown that the connection between high TG and DR is still a topic of debate. It has been demonstrated that in DR, elevated TG levels and high blood sugar interact and jointly contribute to the apoptosis of retinal capillaries, thereby worsening the progression of retinopathy [34]. However, there is insufficient proof of the relationship between high TG and PDR, and after conducting a multivariate analysis, TG levels were not found to be an independent predictor of progression to PDR [35]. In a study on MR, Sobrin et al. discovered a weak correlation between TG and the risk of severe DR (*p* = 0.044) [36]. However, Li et al. concluded no causal association between TG and DR [37]. Our study concluded that TG is causally associated with DR and has a protective effect, although this effect was not significant. This contradicts the traditional view, but it makes sense. Unlike the conventional classification, our exposures are novel high‐resolution lipids classified according to the ratio of carbon to hydrogen, as well as the type, number, and position of the chemical group. This reduces horizontal pleiotropy to some extent. Interestingly, cross‐sectional analyses revealed a negative correlation between multiple TGs and DR progression (TG 50:1, 50:2, 14:0/16:0/18:1, and 50:3) [38]. Patients with mild or moderate NPDR had significantly lower long‐chain aliphatic acid (C ≥ 14), DG, and TG at the lipid level compared to patients without retinopathy [33].

The effect of TG (50:3) on DR may be mediated, in part, by IL‐10 and SLAMF1. IL‐10 is a potent anti‐inflammatory factor that plays a crucial role in promoting an anti‐inflammatory environment in early DR [39]. It promotes macrophage conversion to an anti‐inflammatory phenotype (M2 type), attenuates blood‐retinal barrier disruption, and reduces vascular leakage [40]. In addition, IL‐10 secretion by unstimulated peripheral blood mononuclear cells was significantly increased in patients with NPDR developing from type 1 diabetes [41]. However, as DR develops into PDR, the gradual increase of pro‐inflammatory factors such as IL‐6 and IL‐8 decreases IL‐10 instead. The decrease in IL‐10 secretion may contribute to neovascularization in PDR [41, 42]. We suggest that the early increase in IL‐10 secretion may be a compensatory immune mechanism to protect the retina. As DR progresses, the deterioration of immune function leads to a pro‐inflammatory effect more than an anti‐inflammatory effect. SLAMF1 (also known as CD150) is a member of the signalling lymphocyte activation molecule family, which is mainly expressed in activated T cells, B cells, and macrophages and plays an important role in the regulation of immune homeostasis [43]. Research has shown that autoimmune diabetic mice have defects in SLAM signalling. This deficiency leads to the inability of natural killer T (NKT) cells to differentiate into the IL‐4/IL‐10‐secreting NKT2 phenotype. As a result, normal islet cells become vulnerable to attack by T cells [44]. At the genetic level, differences in methylation at the SLAMF1 (rs3129055) gene locus were strongly associated with individual glucose homeostasis in a large‐scale epigenome‐wide association study (EWAS) [45]. In Indians, the expression of variant‐rs11265455‐SLAMF1 was associated with type 2 diabetes [46]. This indicates that SLAMF1 may play a crucial role in diabetic immune metabolism. However, it remains uncertain whether this immune disorder is linked to the early stage of DR. In conclusion, our findings support that IL‐10 and SLAMF1 may be key targets for early interventions in DR, and we look forward to more longitudinal cohort studies and experimental studies to elucidate the underlying mechanisms.

As a key component of cell membranes, PC is vital for lipid metabolism and cellular function. Notably, DR patients show dysregulation of PC in the blood, with increased alkyl PC and decreased non‐ether PC levels [47]. The role of PC in DR has been relatively understudied. In the early stages of DR, β‐NGF and SIRT2 may be involved. β‐NGF, produced and stored by retinal nerve cells, helps maintain the survival of photoreceptors and prevents their degeneration. In hypoxic conditions, NGF reduces apoptosis and protects mitochondrial membrane potential in human retinal microvascular endothelium [48]. As DR advances, there is an imbalance in NGF and its receptors; levels of mature NGF decrease while pro‐NGF becomes overexpressed, leading to increased retinal inflammation and degeneration of neural and microvascular tissues [49]. These findings suggest that β‐NGF could serve as a dual target for balancing inflammation and providing neuroprotection, which may help slow the progression of DR. SIRT2, a member of the Sirtuins family, has been shown to inhibit the P53/NF‐kB pathway, the inflammatory vesicle NLRP3, and oxidative stress induced by high glucose levels, thereby reducing vascular endothelial injury [50]. Currently, the detection of SIRT2 is emerging as a potential non‐invasive method for the early detection or pre‐screening of DR, presenting a promising strategy for early intervention in this condition [51].

When DR progresses to PDR, the influence of PC on DR is partly mediated by TNFB, also known as lymphotoxin‐alpha (LTA). TNFB is a cytotoxic factor that is produced by activated lymphocytes and exists in both secreted and membrane‐bound forms. It is essential for the development of the immune system, the inflammatory response, and the fight against infections and autoimmune diseases [52]. Research indicates that susceptibility to DR is closely associated with certain polymorphisms of the LTA gene, such as the LTA T60N C → A polymorphism and the 15‐repeat genotype of LTA, along with the LTA NcoI genotype [53, 54]. However, there is no significant association with the 804C/A and 252A/G polymorphisms of the LTA gene [55]. This suggests that the LTA gene may play a crucial role in the development of DR through inflammatory and immunoregulatory mechanisms. These findings provide a theoretical foundation for developing anti‐inflammatory therapies that target the LTA pathway. Future studies are necessary to further clarify the molecular mechanisms involved and to facilitate the transition from genetic testing to precision therapy, ultimately improving the visual prognosis for patients with DR.

Our study found that a specific type of SE (27:1/14:0) is linked to a lower risk of DR. At the same time, it has been suggested that high cholesterol is associated with microvascular complications in patients with diabetes [56]. SE can effectively reduce serum LDL cholesterol levels [57], which may be one of the mechanisms by which SE exerts a protective effect on DR. Additionally, our study indicates that this process may involve LAP TGF‐β1, a precursor of TGF‐β1 [58]. In humans, three forms of TGF‐β exist, and the L10P polymorphism in the TGFβ1 gene may offer potential protection against DR [59]. Interestingly, compared with those in healthy controls, reduced levels of TGF‐β1 are observed in the late NPDR/PDR stages. Dysregulated TGF‐β signalling results in endothelial mesenchymal transition and altered vascular morphology, leading to blood‐retinal barrier dysfunction [60].

## 5 Advantages and Limitations

Our study has several advantages. First, we confirmed the connection between the whole lipidome and DR for the first time using MR analysis. Second, we confirmed the role of 91 inflammatory factors in the lipidome on DR. Third, we analysed DR according to different stages to ensure that our conclusions can be more accurately applied to specific populations.

However, our study has several limitations. First, the effects of genetic variants on most risk factors are usually negligible, so they can explain only a tiny fraction of the variation. This could cause the MR analyses to have low statistical power and false‐negative results. The NPDR database is from the R7 version, which lags behind the most recent databases. Our subjects were of European origin, and the results do not apply to Asian populations, which may lead to biased results and affect the generalisability of the findings. Additionally, the biological functions of the selected SNPs and associated diseases or behaviours have not been fully elucidated in current studies. Moreover, although we discuss the mediating role of different inflammatory factors between lipids and DR, the exact mechanisms by which different lipids affect DR remain to be further investigated.

This study thoroughly examines the cause‐and‐effect relationship between lipidome, inflammatory factors, and DR. We have identified numerous new lipids that protect against DR and could serve as novel biomarkers for DR. Our research highlights the significance of inflammatory factors in elucidating the underlying mechanisms linking lipids and DR. Additionally, our findings provide new perspectives on lipidomic‐based treatments and cytokine‐targeted interventions for DR.

## Author Contributions

**Yuxin Sun:** conceptualization (equal), data curation (lead), formal analysis (equal), methodology (equal), software (lead), visualization (lead), writing – original draft (equal), writing – review and editing (equal). **Ziran Zhang:** conceptualization (equal), writing – original draft (equal), writing – review and editing (equal). **Zejun Chen:** conceptualization (equal), data curation (supporting), investigation (lead), supervision (equal). **Zhengran Li:** formal analysis (equal), visualization (supporting). **Zijin Wang:** methodology (equal), software (supporting). **Fanye Wu:** validation (equal), writing – review and editing (equal). **Xinyu Ma:** validation (equal), visualization (supporting). **Shaoyu Wang:** investigation (supporting), supervision (equal). **Mingzhe Cao:** project administration (equal), supervision (equal). **Guoguo Yi:** conceptualization (equal), project administration (equal), supervision (equal), writing – review and editing (equal). **Min Fu:** conceptualization (equal), project administration (equal), writing – review and editing (equal).

## Ethics Statement

All of the data used in this study was obtained from previously published research and publicly available online databases. Therefore, no further ethics approval or informed consent is required.

## Consent

The authors have nothing to report.

## Conflicts of Interest

The authors declare no conflicts of interest.

## Supporting information


Tables S1‐S4

## Data Availability

All data associated with this study are present in the paper or the Additional files. The GWAS summary data for 179 lipid species can be downloaded using the accession numbers GCST90277238–GCST90277416 from GWAS catalogue (https://www.ebi.ac.uk/gwas/downloads). The statistics for DR and PDR were extracted from the ninth version of the FinnGen Biobank (https://r9.risteys.finngen.fi/), and the data of NPDR were acquired from the seventh edition of the FinnGen Biobank (https://r7.risteys.finngen.fi/). Genetic data for 91 circulating inflammatory proteins were downloaded from the GWAS catalogue (https://www.ebi.ac.uk/gwas/downloads) using accession codes GCST90274758–GCST90274848.
